# Bifidobacterium breve predicts the efficacy of anti‐PD‐1 immunotherapy combined with chemotherapy in Chinese NSCLC patients

**DOI:** 10.1002/cam4.5312

**Published:** 2022-10-07

**Authors:** Honghui Zhao, Dan Li, Jiayin Liu, Xinliang Zhou, Jing Han, Long Wang, Zhisong Fan, Li Feng, Jing Zuo, Yudong Wang

**Affiliations:** ^1^ Department of Medical Oncology The Fourth Hospital of Hebei Medical University Shijiazhuang China

**Keywords:** biomarker, chemotherapy, gut microbes, immunotherapy, non‐small cell lung cancer

## Abstract

**Background and Purpose:**

Gut microbes play an important role in the occurrence of lung cancer, immunotherapy, and chemotherapy. In this study, we analyzed the characteristics of gut microbes in patients with lung cancer and investigated the effect of gut microbes on anti‐PD‐1 therapy combined with chemotherapy.

**Methods:**

Fecal samples from 21 non‐small cell lung cancer (NSCLC) patients and 22 healthy volunteers who were treated in the Fourth Hospital of Hebei Medical University from 2019 to 2021 were collected. DNA was extracted from all samples, and the V3‐V4 region of the bacterial 16S rRNA gene was PCR‐amplified using the Illumina sequencing platform, and R language was used for data analysis.

**Results:**

There were significant differences in the Beta diversity and metabolic pathways of gut microbes between NSCLC patients and healthy individuals (*p* < 0.05). Bifidobacterium, Escherichia, and Sarterella were significantly enriched in patients with clinical benefit response (*p* < 0.05), and these three bacteria had certain predictive value for clinical benefit. Patients with Bifidobacterium breve had significantly longer median progression‐free survival (mPFS) compared with patients with no detectable Bifidobacterium breve feces at baseline (106 days vs. NR, *p* < 0.001). Multivariate COX analysis showed that the presence of B.breve was an independent good prognostic factor affecting the PFS of patients receiving combination therapy (*p* < 0.05).

**Conclusion:**

The clinical efficacy of anti‐PD‐1 therapy combined with chemotherapy in Chinese advanced NSCLC patients is closely related to the gut microbiota, and Bifidobacterium breve may be a potential biomarker to predict the efficacy of immune‐combined chemotherapy.

## Graphical abstract

The effect of intestinal microbiota on PD‐1 immunotherapy combined with chemotherapy in Chinese NSCLC patients remains unclear. Stool samples were collected from patients with NSCLC and healthy volunteers for 16S rRNA V3‐4 sequencing analysis. These results suggest that the clinical efficacy of PD‐1 immunotherapy combined with chemotherapy is affected by some specific intestinal microbiota, and Bifidobacterium brevis may be a potential prognostic biomarker.

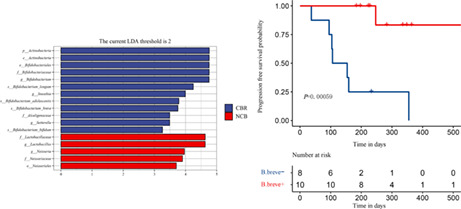

## 1 INTRODUCTION

Lung cancer has extremely high morbidity and mortality in China, and non‐small cell lung cancer (NSCLC) accounts for 85% of the total lung cancer cases.^1^ Immune checkpoint inhibitors (ICIs) targeting anti‐programmed cell death protein 1 (PD‐1) or its ligand (PD‐L1) have significantly changed the treatment and management of locally advanced and advanced NSCLC.^2^, ^3^, ^4^ However, the lack of neoantigen‐specific T cell infiltration and active expression of drug resistance signaling pathways, only 20% of patients with advanced NSCLC benefit from treatment, while up to 50% of patients experience treatment‐related adverse events.^5^, ^6^ Considering the high cost of drugs, limited benefit population, and potentially serious side effects, it is important to explore biomarkers to select patients with advanced NSCLC who may benefit from ICIs therapy. “Gut‐lung axis microecological regulation” has confirmed that gut microbes are closely related to lung cancer,^7^ and play an important role in affecting the efficacy of lung cancer immunotherapy and chemotherapy.^7^, ^8^, ^9^, ^10^

Intestinal microbes are microbial colonies colonized in the intestinal tract and are the main components of human microbial colonies. They are huge in number and complex in diversity, about 10 times the number of human genes. Gut microbes have a significant impact on the metabolism, endocrine, nervous system and immune system of the human body. The composition and diversity of gut microbes are affected by various factors such as environment, genetics, race, age, gender, and survival characteristics. Gut microbiome affects lung immunity and microbiome stability. The gut microbiota and its metabolites can enter the bloodstream through a compromised gut barrier and lead to a long‐term chronic inflammatory state in the body. At the same time, this imbalance of body homeostasis leads to the impaired ability of the immune system to remove damaged and senescent cells, further promoting inflammation and forming a carcinogenic environment, which leads to the occurrence and development of cancer.^11^, ^12^

Multiple studies have suggested that gut microbes are prognostic markers for NSCLC chemotherapy and ICIs immunotherapy. Chemotherapy is an irreplaceable basic treatment for lung cancer. A study found that the use of penicillin, cephalosporin, macrolide and quinolone antibiotics would affect the efficacy of platinum drugs and increase the risk of human lung cancer. The immune responses of specific memory Th1 cells of Coccus and Pasteurella enterica can selectively predict progression‐free survival (PFS) in elderly patients with lung cancer after chemotherapy^13^; In another study, lung cancer patients treated with platinum‐based chemotherapy were specifically identified with an increased number of Enterococcus hela, and suggested better chemotherapy efficacy and prognosis.^14^ Another study further explored the association between intestinal microflora in patients with locally advanced or advanced lung cancer (including 45 NSCLC patients) and the efficacy and survival benefit of first‐line chemotherapy, and found that Streptococcus mutans and Enterococcus caseosa associated with better chemotherapy results, while Leuconostoc lactis and Eubacterium siraeum are gut microbial markers of poor response to first‐line chemotherapy in lung cancer patients.^15^ The approval of immune checkpoint inhibitors in China has also significantly changed the treatment strategy and prognosis of lung cancer patients. Studies by Katayama and others showed that the higher the content of Lactobacillus and Clostridium in the intestinal flora of patients with lung cancer, the better the immune efficacy.^16^ Patients with higher intestinal microbiota diversity are more sensitive to PD‐1 antibody therapy and a. muciniphila has been shown to be significantly associated with ICIs efficacy. Microbiota sterilization of Lewis lung cancer mice with antibiotics Oral supplementation of A.muciniphila ICIs may restore the anticancer effect.^17^ In follow‐up metagenomics analysis studies to further prospectively validate the predictive value of fecal Akk, Akk relative abundance may be a predictor of objective response in advanced NSCLC patients receiving first‐ and second‐line ICI therapy and beyond 12 months biomarkers of survival, in multivariate analysis, baseline fecal Akk was associated with increased objective response rate and overall survival independent of PD‐L1 expression, antibiotics and performance status.^18^ Similarly, other studies have suggested that the diversity of gut microbiota is related to the good response of nivolumab to NSCLC, and the regulation of gut microbiota before treatment may provide a feasible method to improve the efficacy of ICIs immunotherapy.^19^ These studies have shown that there is a correlation between intestinal microbiome and lung cancer treatment, but there are few studies on ICIs immunotherapy combined with chemotherapy.

This study mainly compared the difference between the intestinal flora of NSCLC patients and healthy people, and further explored the characteristics of intestinal flora of NSCLC patients in China, and searched for possible marker species for the occurrence of lung cancer patients. In this study, we mainly performed 16S sequencing on fecal samples of NSCLC patients who received anti‐PD‐1 therapy combined with chemotherapy to explore whether there were significant changes in the intestinal flora of NSCLC patients after the combination therapy. Further, through in‐depth study on the relationship between the characteristics of patients' baseline stool samples and their clinical efficacy, we try to find potential biomarkers for clinical benefit, providing a new direction for the screening and expansion of clinical benefit population and the clinical use of microbial agents.

## 2 MATERIALS AND METHODS

### 2.1 Patients

The selection of research subjects was based on the guidelines of the International Union for Cancer Control. From October 1, 2019 to December 31, 2021, 22 healthy volunteers and 21 patients in the NSCLC group who received immune checkpoint inhibitors combined with chemotherapy in the Medical Oncology Department of the Fourth Hospital of Hebei Medical University were included. All study subjects or guardians must provide written informed consent before participating in the study. All studies were performed under the guidance and permission of the Declaration of Helsinki and the Clinical Trial Ethics Committee of the Fourth Hospital of Hebei Medical University.

The general information of healthy volunteers and patients with NSCLC, including name, gender, age, BMI, smoking and drinking status, etc., were collected. In addition, clinical data including diagnosis, pathological type, TNM stage, number of metastatic organs, PD‐1 expression and ECOG score were also collected in the NSCLC group. Inclusion criteria: (1) pulmonary tumor detected by chest CT or PET/CT; (2) pathological diagnosis of non‐small cell lung cancer; (3) CT/MRI‐evaluable lesions, and at least one evaluation result (refer to RECISTI. l); (4) The patient can collect stool at least once before or after treatment. Exclusion criteria: (1) patients with known autoimmune diseases; (2) previously received immune checkpoint inhibitor therapy (such as PD‐1/PD‐L1/CTLA‐1) or tumor vaccines; (3) Patients with active pulmonary tuberculosis (4) need high‐dose hormone treatment recently; (5) active brain metastases need treatment; (6) patients with cancerous meningitis.

### 2.2 Fecal sample collection

Fresh, mid‐posterior and internal fecal samples (1 g each) were collected from healthy individuals and immediately frozen in a −80°C freezer. Baseline stool samples and post‐treatment stool samples (1 g each) from patients undergoing anti‐PD‐1 therapy combined with chemotherapy were collected in the same way, and immediately frozen in a −80°C freezer.

### 2.3 Experimental process

The OMEGA Soil DNA Kit (M5635‐02) (Omega Bio‐Tek) was used to extract the genomic DNA of stool samples, the DNA was quantified by Nanodrop, and the quality of DNA extraction was detected by 1.2% agarose gel electrophoresis. Based on the 16S rRNA V3–V4 region of microorganisms, the corresponding primers were designed: F: ACTCCTACGGGAGGCAGCA; R: GGACTACHVGGGTWTCTAAT, and the sample‐specific Barcode sequence was added, and then the rRNA gene V3–V4 fragment was amplified by PCR. The amplification products were purified and recovered by magnetic beads, and the PCR amplification products were quantified by fluorescence. The fluorescent reagent was Quant‐iT PicoGreen dsDNA Assay Kit, and the quantitative instrument was Microplate reader (BioTek, FLx800). According to the fluorescence quantitative results, each sample is mixed in a corresponding proportion according to the sequencing volume requirement of each sample. Illumina NovaSeq sequencing was used to prepare the sequencing library using Illumina's TruSeq Nano DNA LT Library Prep Kit.

### 2.4 Data analysis

Sequence denoising was performed according to the QIIME2 dada2 analysis procedure. Show the specific composition of each sample (group) at different taxonomic levels of species to understand the overall overview. The alpha diversity level of each sample was assessed based on the distribution of ASVs across different samples. At the ASV level, the distance matrix of each sample is calculated, and through a variety of unsupervised sorting and clustering methods, the multivariate analysis of variance (PERMANOVA) test method is used to measure the Beta diversity between different samples (groups). Sexual differences and their significance. At the taxonomic composition level, through various unsupervised and supervised sorting, clustering and modeling methods, the Wilcoxon test statistical test method is used to further measure the differences in species abundance composition between different samples (groups), and try to find marker species.

All continuous variables are expressed as mean ± standard deviation (SD). The comparison between the two groups was performed using the independent sample *t* test, and the categorical variable was performed using the χ^2^ test. SPSS 24.0 was used for statistical analysis. Logistic regression model was used to establish the intestinal microbiota model to help predict the efficacy of PD‐1 immunotherapy combined with chemotherapy for NSCLC, and the ROC curve was drawn using R language (version 4.1.2). Referring to the known microbial genome data, the PICRUSt2 software was used to analyze the prediction of sample functional abundance based on the abundance of 16S rRNA gene sequencing sequences. The research mainly predicted the 16S rRNA gene sequence in the KEGG functional database. According to the obtained functional units and the normalized pathway/group abundance table, use the R language to obtain the abundance value of the metabolic pathway; based on the results of abundance statistics, STAMP software (version 2.1.3) was used to analyze metabolic pathway differences and try to find out metabolic pathways with significant differences between groups. In addition, we used R language (version 4.1.2) to perform Cox regression and KM survival analysis (the Log Rank test) to evaluate the relationship between the gut microbiota and the clinical median survival time of patients. *p* < 0.05 was considered statistically significant.

## 3 RESULTS

### 3.1 Difference in intestinal flora between NSCLC patients and healthy individuals

In this study, 21 patients constituted the NSCLC group, and 22 healthy volunteers constituted the healthy control group (HC), including 18 male patients and 3 female patients in the NSCLC group, and 8 healthy male patients and 14 healthy female patients in the HC group. The gender, age, BMI, smoking and drinking habits of the two groups are summarized in Table 1. Except for the gender difference (*p <* 0.05), other observation variables were statistically different between the two groups of subjects. In the NSCLC group, there were 11 patients with adenocarcinoma and 10 with squamous cell carcinoma; 6 patients with tumor TNM stage III, 15 with IV stage. In the NSCLC group, 12 patients received anti‐PD‐1 therapy combined with chemotherapy as first‐line therapy, and the remaining 9 patients started therapy after failure of previous therapy.

**TABLE 1:** Characteristics of the NSCLC patients group and healthy control group

	HC group (*n*=22)	NSCLC group (*n*=21)	*p* value
Age (Mean±SD, year)	33.2±5.7	61.3±9.6	0.130
Gender (male/female)	8/14	18/3	0.007
BMI (kg/㎡)	23.6±2.5	23.7±2.9	0.479
Smoking [*n* (%)]	8 (36.4%)	12 (57.1%)	0.172
Drinking [*n* (%)]	9 (40.9%)	11 (52.4%)	0.451
Pathological classification [*n*(%)]			
Adenocarcinoma		11 (52.4%)	
Squamous carcinoma		10 (47.6%)	
Tumor stage [*n* (%)]			
III		6 (28.6%)	
IV		15 (71.4%)	
Lines [*n* (%)]			
1		12 (57.1%)	
>1		9 (42.9%)	

*Note*: The continuous variables are listed as mean ± SD.

**FIGURE 1:**
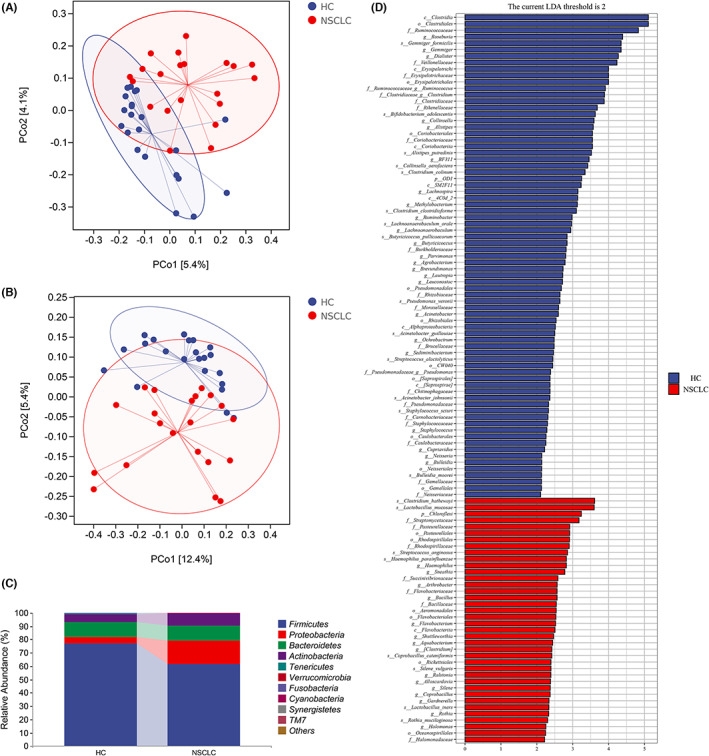
Analysis of intestinal flora characteristics of NSCLC patients and healthy people. (A) PCoA analysis of inter‐group Beta diversity based on Jaccard distance algorithm (PERMANOVA, *p* = 0.001) Blue represents HC group sample; Red represents the NSCLC group samples; (B) PCoA analysis of inter‐group Beta diversity based on unweighted UniFrac distance algorithm (PERMANOVA, *p* = 0.002); (C) Species composition analysis was performed at phylum level, and the relative abundance of the top 10 species was displayed by means of the samples within the group. (D) Histogram of LDA value distribution of species with significant differences, showing robust differential species between HC group and NSCLC group analyzed by LEfSe, by Wilcoxon test, *p* < 0.05. HC, healthy control group; NSCLC, non‐small cell lung cancer group.

Comparing the alpha diversity of the intestinal flora between the NSCLC group and the HC group, the analysis found that the alpha diversity of the samples in the two groups was not statistically significant in the correlation analysis of richness, diversity and evenness (Figure S1A; *p* > 0.05). PCoA analysis based on Jaccard and unweighted UniFrac distance algorithm was performed on the two groups of samples, and it was found that there was a significant difference in beta diversity between patients and healthy people (*p* = 0.001 and *p* = 0.002; Figure 1A,B). Species composition analysis showed that the two groups of patients had similar species composition at the phylum level, including Firmicutes, Bacteroidetes, Proteobacteria, and Actinobacteria, all of which were dominated by Firmicutes (Figure 1C). Further, the LEfSe analysis was used to directly perform differential analysis on all taxonomic levels at the same time to find robust differential species between groups, namely biomarkers. Compared with the NSCLC group, the HC group was significantly enriched in different taxonomic levels such as Clostridia, Clostridiales, Ruminococcaceae, Roseburia, and Gemmiger formicilis, and so on. Compared with the HC group, the different species in the NSCLC group were Clostridium hathewayi, Lactobacillus mucosae, Chloroflexi, Streptomycetaceae and other different taxonomic levels of gut microbes (*p* < 0.05) (Figure 1D).

The study further used the sequencing sequence abundance to perform functional prediction in the KEGG functional database to analyze the metabolic pathways of the NSCLC group and the HC group. From the metabolic pathway abundance statistics map, there are 34 related metabolic pathway gene families in the two groups of gut microbes. From the perspective of relative abundance, it is mainly related to metabolism, such as Carbohydrate metabolism, Amino acid metabolism, Metabolism of cofactors and vitamins, Metabolism of terpenoids and polyketides, Lipid metabolism, etc. (Figure S2). After obtaining the abundance data of metabolic pathways, the study tried to find out the metabolic pathways with significant differences between the NSCLC group and the HC group, and evaluated the significant differences through statistical tests. The results indicated that a total of 35 KO functional groups were significantly different in abundance between the two groups (Figure S3).

### 3.2 Changes of intestinal flora in NSCLC patients before and after anti‐PD‐1 therapy combined with chemotherapy

**FIGURE 2:**
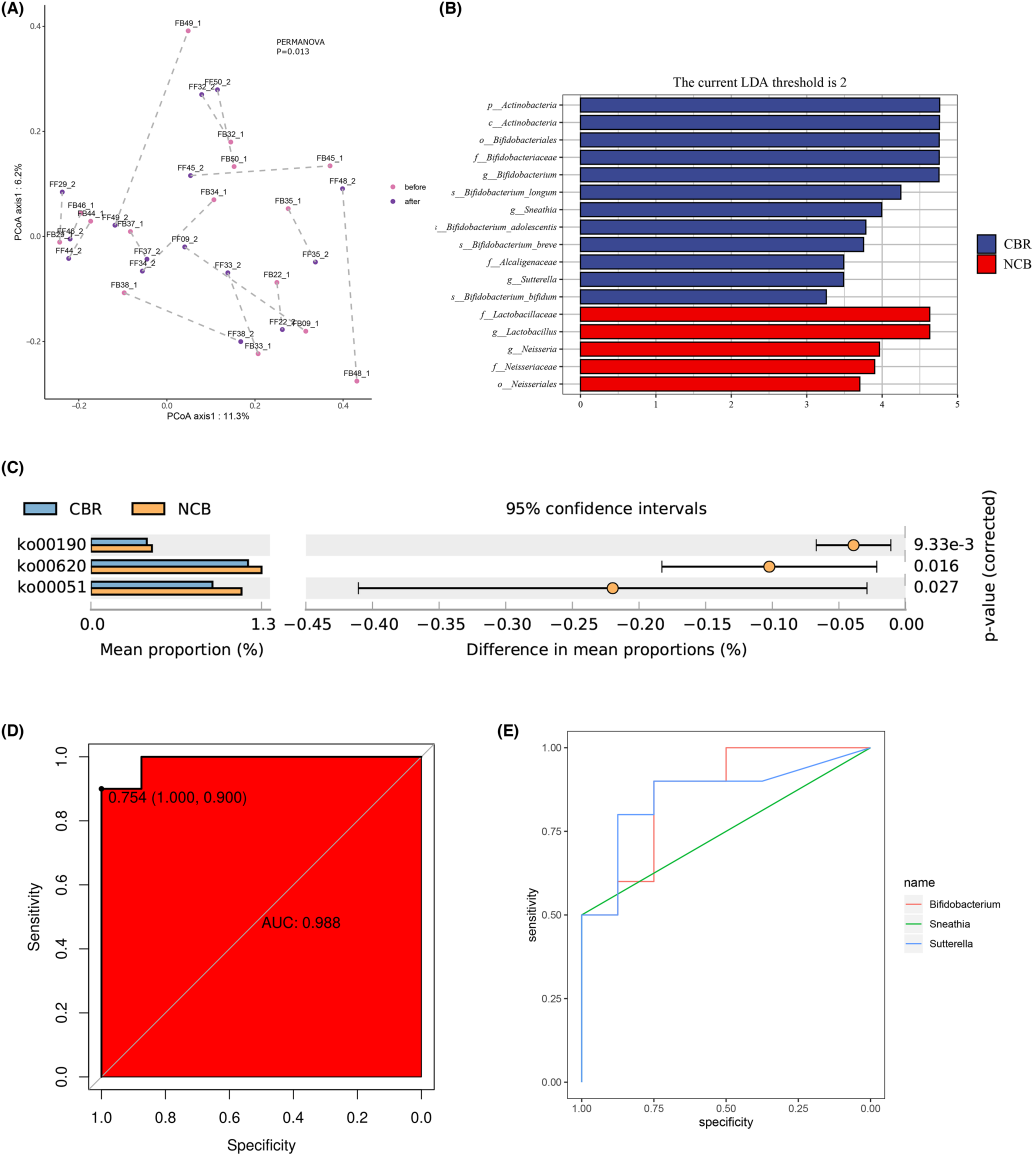
Correlation between intestinal flora and anti‐PD‐1 therapy combined with chemotherapy. (A) PCoA analysis of Beta diversity between individuals before and after combined therapy based on Bray‐Curtis distance algorithm (PERMANOVA, *p* = 0.013); (B) LEfSe analysis of species differences and marker species(Wilcoxon rank testing, *p*<0.05). Histogram of LDA value distribution of significantly different species, showing species significantly enriched in CBR group and NCB group and their significance *p* < 0.05; (C) Metabolic pathway differences between CBR and NCB groups(welch's t‐test, all *p* < 0.05). (D) Logistic regression prediction model was used to analyze the predictive ability of three genera with significant differences in fecal intestinal microorganisms between CBR group and NCB group. (E) ROC curve analysis of Bifidobacterium, Sneathia, and Sutterella for predicting clinical benefit. CBR, clinical benefit response, NCB, no‐clinical benefit.

To explore the effect of anti‐PD‐1 immunotherapy combined with chemotherapy on the gut microbiota of NSCLC patients, this study analyzed paired stool samples from 15 NSCLC patients before treatment (before group) and after treatment (after group). The results showed that there was no significant change in the alpha diversity and beta diversity of intestinal flora before and after combined treatment (Figure S1B,C) (*p* > 0.05), and the alpha diversity of the samples did not change with the leveling depth increased and gradually increased (Figure S1D). However, after fully considering individual differences, PCoA analysis based on the bray‐curtis distance showed that there was a significant difference in the changes of gut microbiota between individuals with lung cancer before and after combined treatment (*p* = 0.013) (Figure 2A). According to the prediction of functional potential, compared with the combination of immunotherapy and chemotherapy, the six metabolic pathways of photosynthesis ‐ antenna proteins, tropane, piperidine and pyrimidine alkaloid biosynthesis, tryptophan metabolism, styrene degradation, photosynthesis and geraniol degradation before the combination therapy were more significant (Figure S4A).

### 3.3 The clinical efficacy of anti‐PD‐1 therapy combined with chemotherapy in NSCLC patients is closely related to the intestinal flora

Based on the obvious differences in the changes of gut microbiota among the treated patients, this study further explored the effect of anti‐PD‐1 immunotherapy combined with chemotherapy on the clinical efficacy of patients. Because 3 patients were lost to follow‐up or died after combined treatment, the imaging review was not completed, and the remaining 18 NSCLC patients were evaluated for clinical efficacy. The clinical benefit response (CBR) group was defined as complete response (CR), partial response (PR) or stable disease (SD) duration ≥6 months according to the RECIST 1.1 standard response evaluation, and the efficacy evaluation was stable disease duration <6 months or progressive disease (PD) was defined as the non‐clinical benefit (NCB) group. Among them, there were 10 patients in the CBR group and 8 patients in the NCB group. LEfSe analysis found that Actinobacteria were significantly increased in the CBR group at the phylum and class levels. At the order level, Bifidobacteriales were significantly enriched in the CBR group, while Neisseriales were higher in the NCB group. At the family level, Lactobacillaceae and Neisseriaceae were significantly enriched in NCB group, while Bifidobacteriaceae and Alcaligenaceae were enriched in CBR group. At the genus level, Lactobacillus and Neisseria increased in NCB group, while Bifidobacterium, Sneathia, Sutterella in CBR group Genus increases. It is worth noting that compared with the NCB group at the species level, Bifidobacterium longum (B.longum), Bifidobacterium adolescentis (B.adolescentis), Bifidobacterium bifidum (B.bifidum) and Bifidobacterium breve (B.breve) were significantly enriched in the CBR group (Figure 2B). In conclusion, there was a significant enrichment of bifidobacteria in the intestinal flora of the CBR group. Functional potential prediction showed that compared with CBR group, NCB group had significantly higher levels of oxidative phosphorylation, pyruvate metabolism, and fructose and mannose metabolism (Figure 2C).

Considering that 16S is more accurate at the genus level, the study established a machine learning model for gut microbes to predict efficacy. To explore the specific gut microbiota that can help predict the efficacy of immunotherapy combined with chemotherapy for lung cancer, we used Logistic regression model to analyze the predictive power of Bifidobacterium, Sneathia, and Sutterella, which were significantly different in fecal gut microbes between.

the CBR group and the NCB group. The results showed that the three microbial genera had high accuracy in predicting combination therapy for NSCLC, with an area under the curve of 0.988, and sensitivity and specificity of 100% and 90%, respectively (Figure 2D). Further ROC curve analysis showed that Bifidobacterium, Sneathia, and Sutterella had high sensitivity and specificity in predicting efficacy (Figure S4B‐D). The results showed that these three intestinal bacteria genera have certain clinical significance in predicting NSCLC immunotherapy combined with chemotherapy (Figure 2E).

### 3.4 Predictive effect of bifidobacteria on the clinical efficacy of anti‐PD‐1 therapy combined with chemotherapy in NSCLC

**FIGURE 3:**
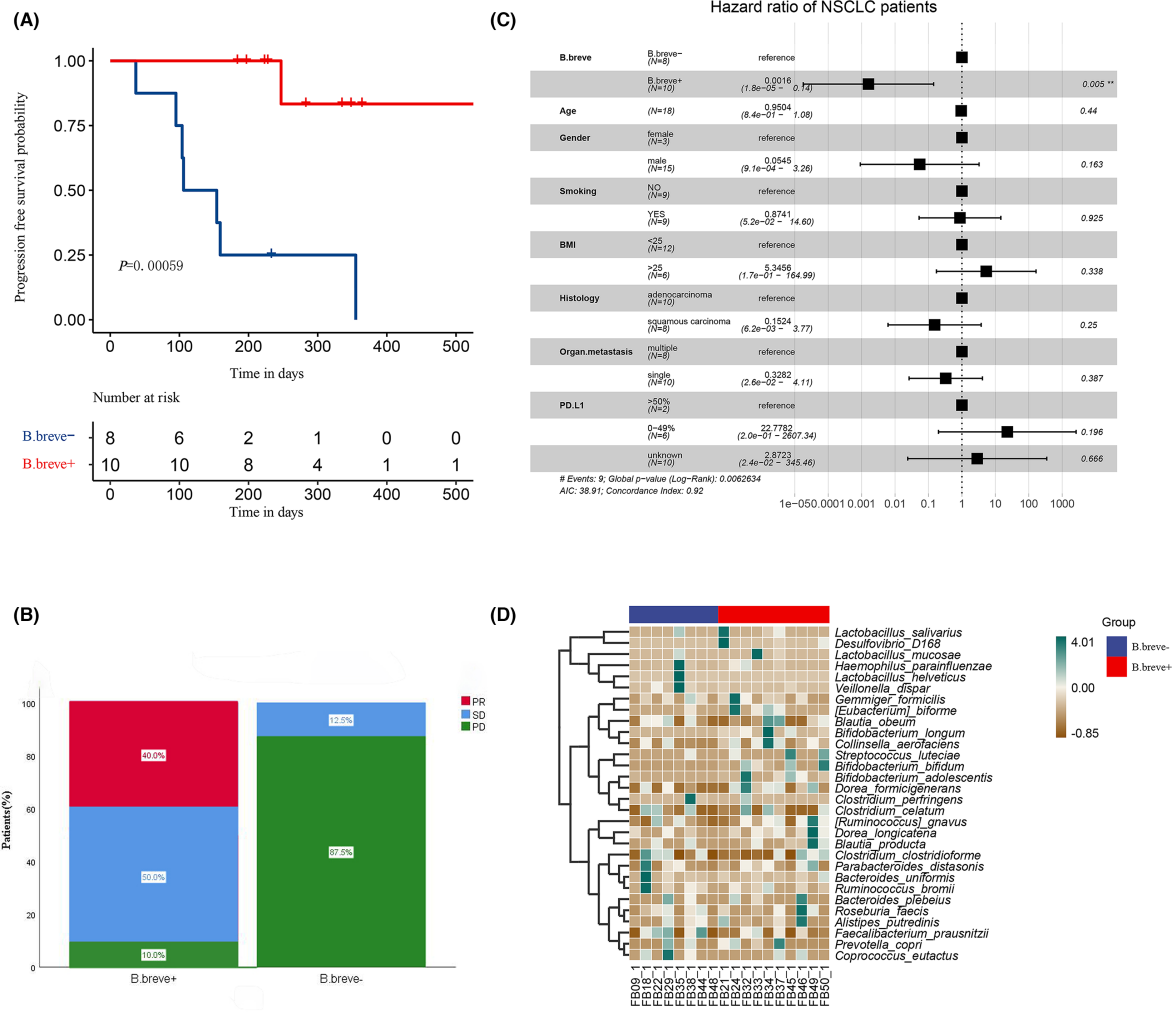
Effect of B.breve on clinical outcomes and species composition. (A) B.breve k‐M survival analysis of patients receiving treatment, (The Log Rank test, *p* = 0.000,59); (B) B.breve+ group and B.Breve ‐ group efficacy evaluation results. Before group: before treatment; After group: after treatment. PD, progressive disease; SD, stable disease PR, partial response; (C) Multivariate COX survival analysis of 18 patients; (D) Heat map of species composition difference between B.breve+ group and B.breve‐ group. Bifidobacterium, Escherichia, and Sarterella were significantly enriched in patients with clinical benefit response (*p* < 0.05), and these three bacteria had certain predictive value for clinical benefit.

To further explore whether B.longum, B.adolescentis, B.bifidum, or B.breve can effectively predict the clinical benefit of patients, survival analysis was performed in this study. Survival analysis was performed based on whether these four bacteria could be detected in baseline stool samples of 18 NSCLC patients. As none of the 17 patients (94%) died, the primary objectives were progression‐free survival (PFS, defined as the time from inclusion to either the first disease progression event according to the RECIST 1.1 criteria or death from any cause, whichever occurred first). The study found that the content of B.breve significantly affected the PFS of patients (*p* < 0.001), and the other three species did not significantly affect the PFS of patients (Figure S5A–C; *p >* 0.05). Specifically, the median progression‐free survival (mPFS) in the B.breve‐containing (B.breve+) group was not reached (NR, 95% CI: NC‐NC), while the mPFS in the no B.breve‐containing (B.breve‐) group was 106 days (95% CI: 37–175) (Figure 3A). Among the 10 patients in the B.breve+ group, 4 patients were evaluated as PR, 5 were SD, and 1 patient was PD; while in the B.breve‐ group, 1 patient was evaluated as SD, and the remaining 7 patients were PD (Figure 3B). For the B.breve+ and B.breve‐ groups in our cohort, the objective response rate (ORR) of the two groups were 0% and 40%, respectively (*p* = 0.043), and the disease control rate (DCR) was 90.0% and 12.5%, respectively(*p* = 0.001).

Multivariate COX analysis showed that age, personality, smoking status, pathological type, organ metastasis and PD‐L1 expression were not independent prognostic factors for PFS (*p* > 0.05). Only the presence of B.breve was an independent good prognostic factor affecting the PFS of patients receiving combination therapy (*p* < 0.05) (Figure 3C). The species composition of patients in B.breve+ group and B.breve‐ group was analyzed, and it was found that among the top 30 species in average abundance, the abundance of B.longum, B. dolescentis and B.bifidum were increased in the intestinal flora of the B.breve+ group (Figure 3D). The difference analysis of metabolic pathways showed that compared with the B.breve‐ group, the B.breve+ group significantly increased the abundance of metabolic pathways, such as cysteine and methionine metabolism, valine, leucine and isoeucine biosynthesis, and C5‐branched dibasic acid metabolism (Figure S6).

## 4 DISCUSSION

Similar to previous studies,^20^ our study once again showed that there were significant differences in the gut microbiota between NSCLC patients and healthy individuals. It is worth noting that there is a significant enrichment of B. adolescentis in the gut of healthy people, and studies have shown that Bifidobacterium has inhibitory effects on the occurrence and development of various tumors. Bifidobacterium mainly exerts this effect in three ways: first, it activates the body's immune system, which is consistent with its immune‐enhancing function, specifically by enhancing macrophages, NK cells, and B lymphocytes to exert tumor‐suppressing effects.^21^, ^22^ Secondly, it affects the biochemical metabolism of the intestinal flora, reduces the production of carcinogens, inhibits the activity of enzymes, reduces the intestinal value, promotes peristalsis, accelerates the elimination of metabolites, and reduces the contact of carcinogens with the intestinal epithelium.^23^ Finally, bifidobacteria can induce apoptosis of tumor cells, thereby maintaining the normal phenotype of the cells and preventing the occurrence of tumors.

The study analyzed the characteristics of intestinal flora in NSCLC patients receiving anti‐PD‐1 immunotherapy combined with chemotherapy. The results showed that the intestinal flora of patients did not change significantly before and after combined treatment, but there were significant differences in the changes of intestinal flora between individuals. This provides a basis for us to further study the relationship between individual clinical efficacy and gut microbiota. Compared with patients who did not benefit from clinical practice, there was a significant enrichment of Bifidobacterium, Escherichia, and Sarterella in the intestinal flora of patients with clinical benefit, which could predict NSCLC patients receiving anti‐PD‐1 Efficacy of treatment combined with chemotherapy.

Bacterial organisms have the ability to replicate and target hypoxic regions of tumors in vivo, whereas typically only 3%–5% of tumor cells are considered to be the growing part, and the remaining 95% of tumor tissue has a hypoxic environment inside. ^24^ As a probiotic, Bifidobacterium has the characteristics of tending to an anaerobic environment. Bifidobacterium longum 105A and 108A strains were given to Lewis lung cancer mice by tail vein injection, the results showed that Bifidobacterium can be implanted into tumors in a targeted manner, while normal tissues such as liver, spleen, and kidney did not grow Bifidobacterium.^25^ In recent studies, the role of specific components produced by gut microbes (including Bifidobacterium) in tumorigenesis and anti‐tumor therapy has gradually attracted widespread attention, especially short‐chain fatty acids (SCFAs). SCFAs, such as acetate, propionate, and butyrate, are produced by microbial fermentation of indigestible fiber by gut flora. SCFAs have the ability to inhibit tumor growth by reversing epigenetic changes by acting as histone deacetylase (HDAC) inhibitors; they can promote cancer cell death by regulating miRNA; by stimulating the expression of cell cycle‐regulated genes p53 and p21, reducing the Expression of anti‐apoptotic Bcl‐2 protein, as well as increased expression of pro‐apoptotic Bax protein, increases apoptosis in some adenomas and cancer cells.^26^, ^27^ SCFAs exert direct anti‐colorectal cancer effects mainly by inhibiting histone deacetylases (HDACs) or binding to SCFAs receptors (G protein‐coupled receptors, GPRs).^28^ The study provided evidence that SCFAs enhanced the effect of chemotherapy, and the combination of propionate and cisplatin enhanced the therapeutic effect of cisplatin.^29^ In contrast, SCFA was associated with significant long‐term benefit in NSCLC patients treated with anti‐PD‐1 nivolumab. Selected patients in the study received calculated doses of nivolumab every 2 weeks until disease progression was detected, and their stool samples were collected for metabolomic analysis of the gut microbiota. The results showed that the gut microbiota of patients who responded to immunotherapy was characterized by high levels of SCFAs, particularly butyrate and propionate.^30^ In another study, solid tumor anti‐PD‐1 efficacy was associated with higher concentrations of SCFAs, in which patients received nivolumab or pembrolizumab for the appropriate duration and frequency. SCFA levels were analyzed in stool and plasma samples from responder and non‐responder patient groups. After analyzing the samples, they found higher SCFA concentrations in the responder group compared to the non‐responders.^31^

The important thing is that this study analyzed that B.breve in the gut significantly affects the mPFS of NSCLC patients receiving anti‐PD‐1 immunotherapy combined with chemotherapy, and B.breve may be an effective biomarker to predict its clinical benefit. It has been shown that B.breve exposure increases the expansion capacity of H2‐Kb SIY complex (KbSIY) reactive cells, resulting in an increase in CD8+ T cells with higher affinity responses and more robust KbSIY cross‐reactivity, while KbSVY responses can target and slow tumor progression.^32^ Animal experiments showed that B.breve can significantly inhibit the growth of transplanted tumors in C3H/HeN mice and upregulate the recruitment of intestinal tumor‐infiltrating lymphocytes (TILs) and dendritic cells (DCs) in the tumor microenvironment, and interleukin 12 (IL‐12) secreted by DCs plays a crucial role in this process.^33^ Oral administration of commensal Bifidobacterium alone can enhance antitumor immunity in vivo.^34^ The revealing of the effect of B.breve may provide a new therapeutic direction for the treatment of Chinese NSCLC patients.

From the prediction of metabolic pathway potential, these gut microbes mainly affect the body's metabolic function. Healthy people are more significant in the metabolic pathways that are beneficial to the decomposition of exogenous substances in the body, prevent tumorigenesis, innate immunity and promote cell apoptosis; while NSCLC tumor patients have complex metabolic changes, especially tumor aerobic sugar Glycolytic changes and alterations in amino acid metabolism. Metabolic difference analysis showed that NSCLC patients received PD‐1 immunotherapy combined with chemotherapy accompanied by significant metabolic changes. Compared with the clinical benefit population, the non‐clinical benefit population has increased oxidative phosphorylation energy metabolism and carbohydrate metabolism, which may be closely related to intestinal bifidobacteria. It has also been shown that the oxidative metabolism of tumor cells is a barrier to PD‐1 immunotherapy, and that mitochondrial oxidative phosphorylation is not only not impaired but more activated in several cancers (pancreatic adenocarcinoma, melanoma, and leukemia).^35^ The combination of radiotherapy and oxidative phosphorylation inhibitors has been proved to be an effective strategy against PD‐1 resistance in NSCLC.^36^

Of course, this study has limitations that cannot be ignored. First, due to the small sample size and short follow‐up time of the study subjects, the OS data were not obtained in the study data; secondly, it is necessary to carry out clinical verification and exploration of the regulatory mechanism on the basis of expanding the sample size. Third, we did not dynamically monitor the changes in the gut microbiota of patients during combination therapy, which may help to more fully understand the correlation between the microbiota and efficacy and survival benefits. Therefore, this project plans to conduct further in‐depth research, including expanding the sample size and adopting a longitudinal monitoring design method; at the same time, constructing an animal model of NSCLC receiving combined drug treatment to study the impact mechanism of intestinal microbes; using metagenomics technology to more accurately analyze the intestinal microbial markers.

## 5 CONCLUSION

There are significant differences in the intestinal flora β diversity and metabolic pathways between NSCLC patients and healthy people, which may be closely related to the occurrence and development of lung cancer. Although the gut microbiota did not change significantly after patients received anti‐PD‐1 immunotherapy combined with chemotherapy, significant changes were still found from an individual perspective. Certain specific gut microbiota have potential correlation and predictive value with the clinical outcomes of combination therapy, including different clinical efficacy and survival benefits. B.breve may be a potential biomarker to predict the survival benefit of NSCLC immunotherapy combined with chemotherapy, providing new research ideas for improving the efficacy of immunotherapy.

## AUTHOR CONTRIBUTIONS

**Honghui Zhao:** Data curation (equal); formal analysis (equal); methodology (equal); project administration (equal); software (equal); writing – original draft (equal); writing – review and editing (equal). **Dan Li:** Investigation (equal); resources (equal). **Jiayin Liu:** Methodology (equal); resources (equal). **Xinliang Zhou:** Software (equal); validation (equal). **Jing Han:** Data curation (equal); formal analysis (equal). **Long Wang:** Data curation (lead); visualization (equal). **Zhisong Fan:** Conceptualization (equal); project administration (supporting); writing – review and editing (equal). **Li Feng:** Resources (equal); writing – review and editing (supporting). **Jing Zuo:** Conceptualization (supporting); supervision (equal); writing – review and editing (equal). **Yudong WANG:** Conceptualization (supporting); project administration (lead); supervision (lead); writing – review and editing (lead).

## FUNDING INFORMATION

This study was supported by the Natural Science Foundation of Hebei Province (H2020206551), the Cancer Research Program of National Cancer Center (NCC2017A30), and the Beijing XIsiKE Clinical Oncology Research Foundation (Y‐MSDPU2021‐0202).

## Supporting information


Figure S1

Figure S2

Figure S3

Figure S4

Figure S5

Figure S6

Appendix S1

## Data Availability

Data available on request from the authors.
