# Supplementary figures and images for: Bifidobacterium breve predicts the efficacy of anti‐PD‐1 immunotherapy combined with chemotherapy in Chinese NSCLC patients
Source: Cancer Med. 2022 Oct 7;12(5):6325–36. doi: 10.1002/cam4.5312 (PMC10028067; doi:10.1002/cam4.5312)

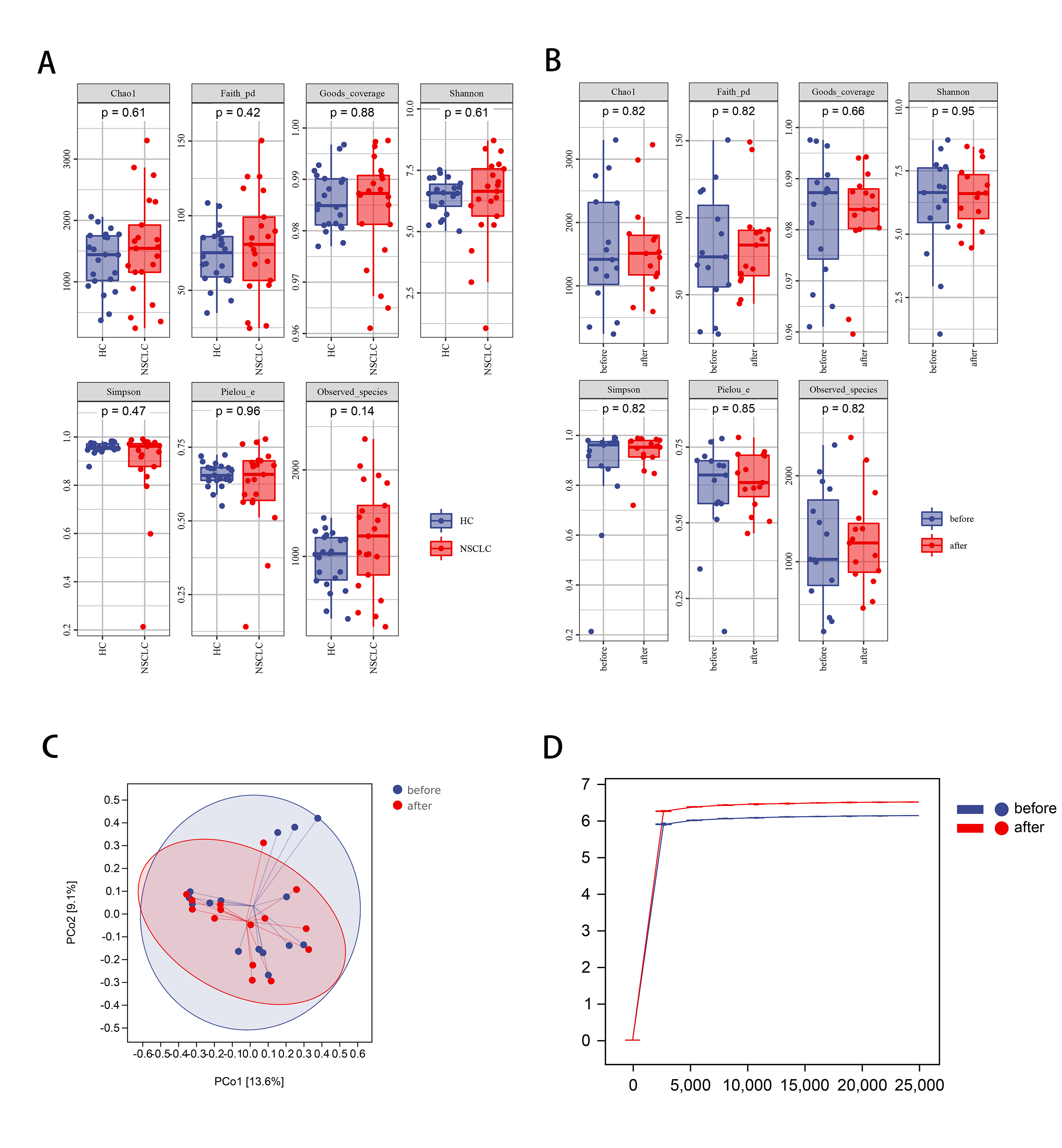

Supplement: Supplementary file 1 — Figure S1 [file CAM4-12-6325-s006.tif]

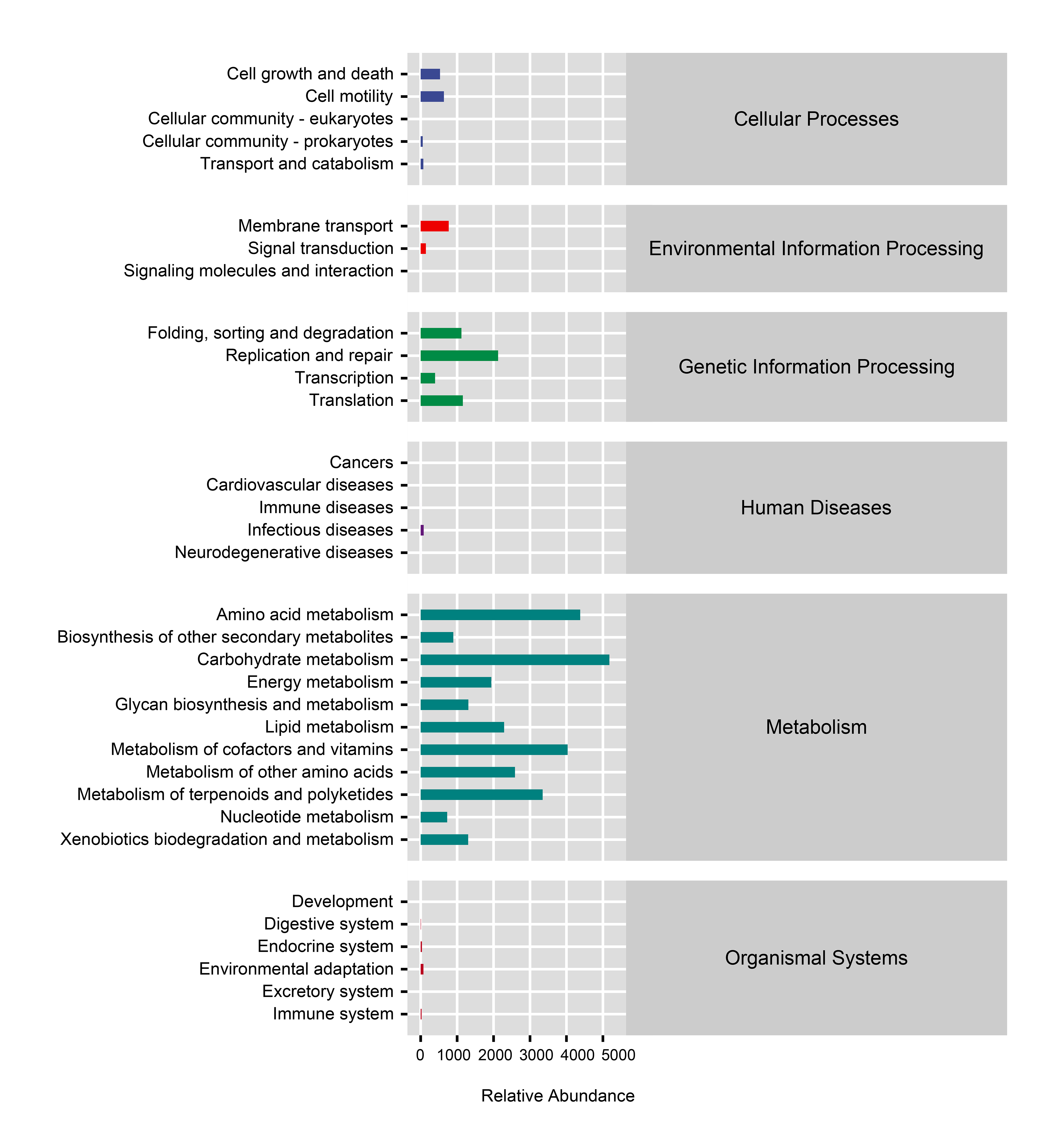

Supplement: Supplementary file 2 — Figure S2 [file CAM4-12-6325-s002.tif]

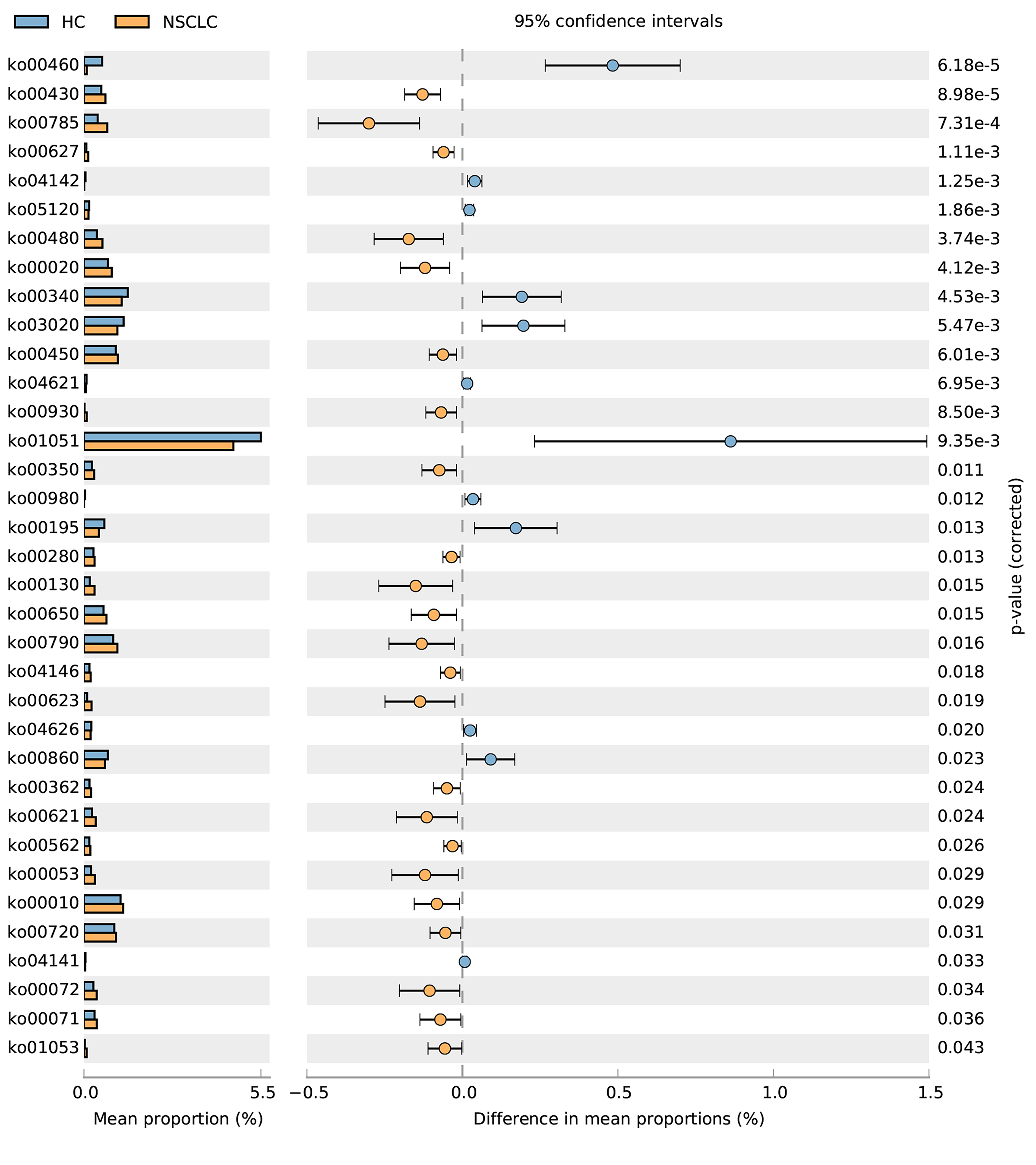

Supplement: Supplementary file 3 — Figure S3 [file CAM4-12-6325-s007.tif]

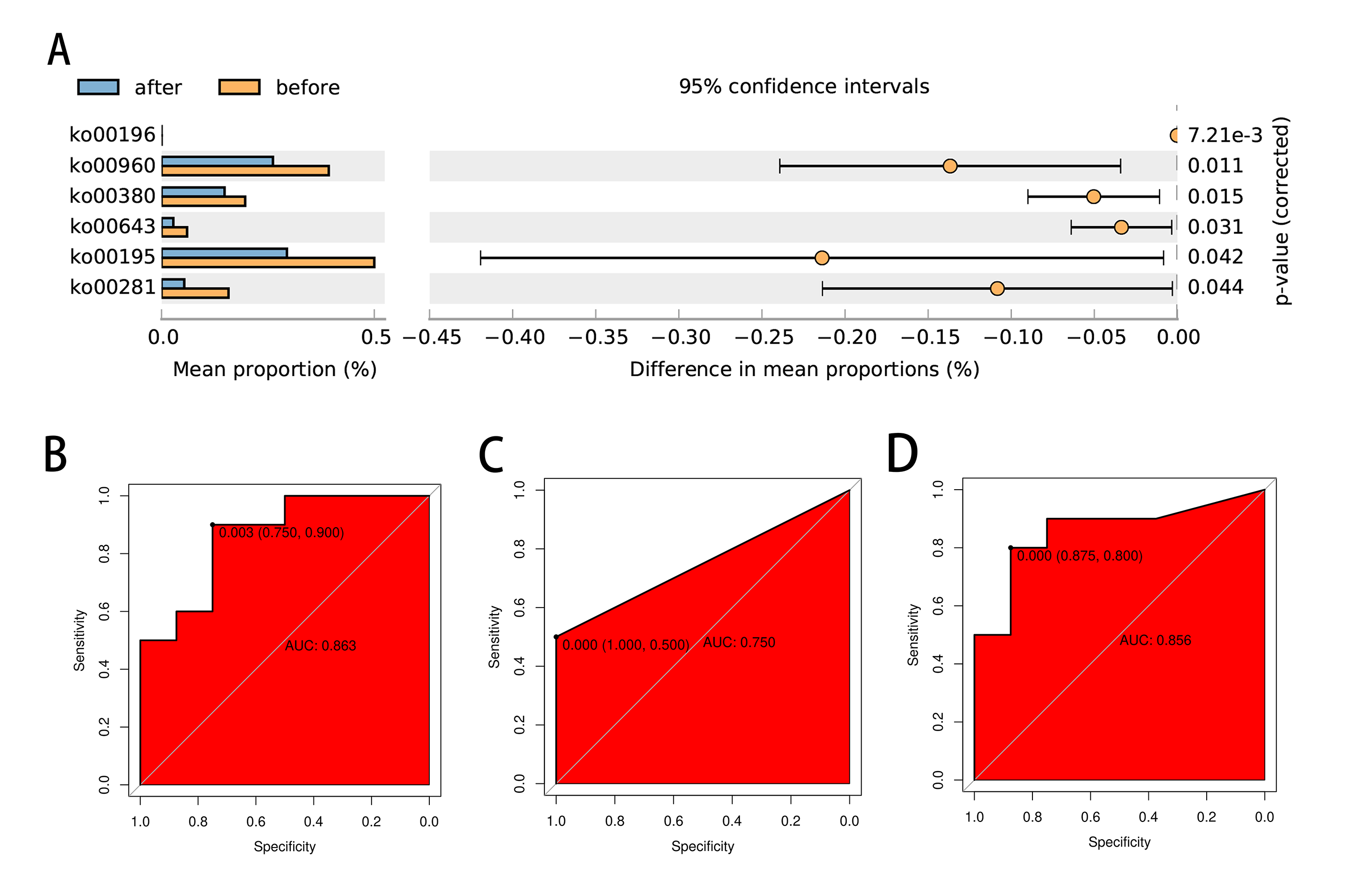

Supplement: Supplementary file 4 — Figure S4 [file CAM4-12-6325-s001.tif]

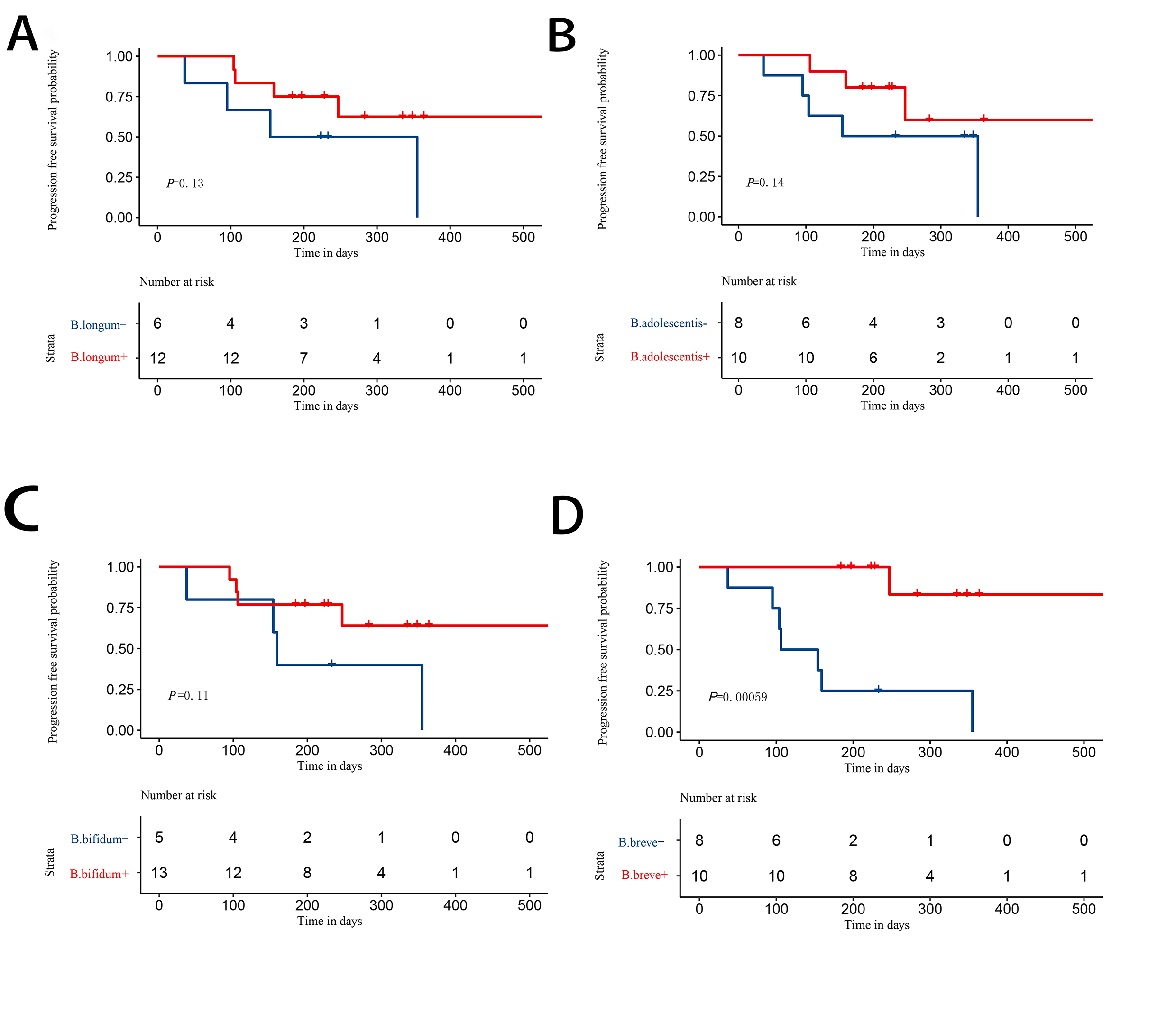

Supplement: Supplementary file 5 — Figure S5 [file CAM4-12-6325-s004.tif]

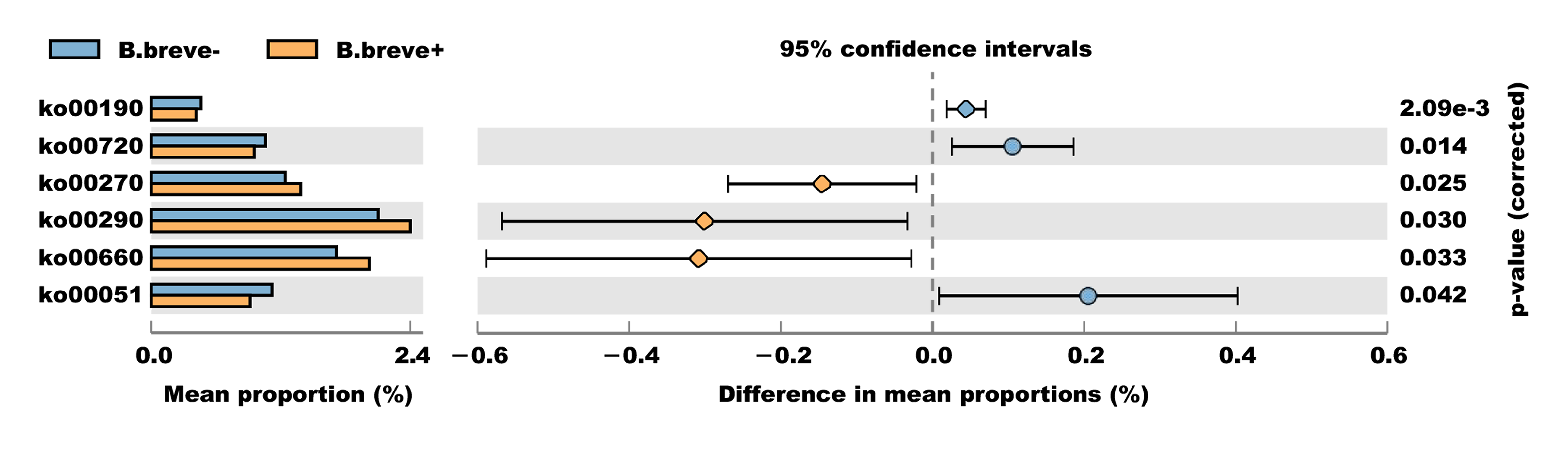

Supplement: Supplementary file 6 — Figure S6 [file CAM4-12-6325-s005.tif]
