# Supplementary material for: Bifidobacterium breve predicts the efficacy of anti‐PD‐1 immunotherapy combined with chemotherapy in Chinese NSCLC patients
Source: Cancer Med. 2022 Oct 7;12(5):6325–36. doi: 10.1002/cam4.5312 (PMC10028067; doi:10.1002/cam4.5312)
Supplement: Supplementary file 7 — Appendix S1 [file CAM4-12-6325-s003.docx]

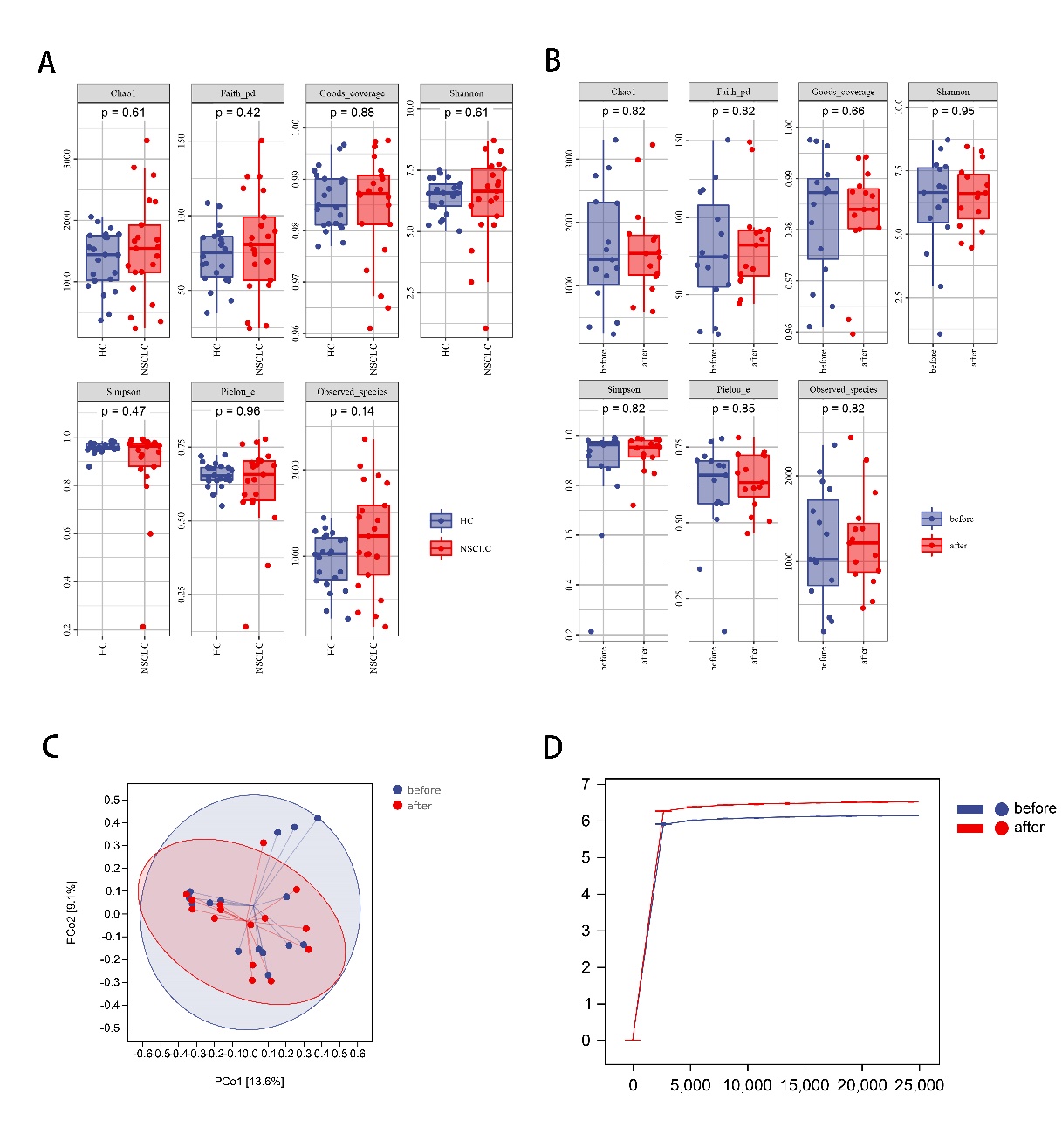


Supplementary Figure 1. Comparative analysis of species diversity. A. Comparison of Alpha diversity between NSCLC group and HC group Chao1 index and Observed Species index were used to characterize richness, Shannon index and Simpson index to characterize diversity, Faith's PD index to characterize evoluence-based diversity, Pielou's Evenness index to characterize evenness and Good's Coverage index showed no significant difference (Kruskal-Wallis rank-sum test and Dunn 'test, *P* > 0.05). B. Comparison of Alpha diversity of 15 patients before and after anti-PD-L1 treatment combined with chemotherapy Chao1, Observed Species, Shannon, Simpson, Faith's PD, Pielou's Evenness, Good's in the two groups There was no significant difference in coverage index (Kruskal-Wallis rank sum test and Dunn 'test, *P* > 0.05). C. PCoA analysis of inter-group Beta diversity based on Bray-Curtis distance algorithm ((PERMANOVA,, *P*=0.881).D. Draw Rarefaction Curve, and sample alpha diversity of before group and after group is sufficiently extracted and leveled. HC: healthy control group; NSCLC: non-small cell lung cancer group. Before group: before treatment; After group: after treatment.


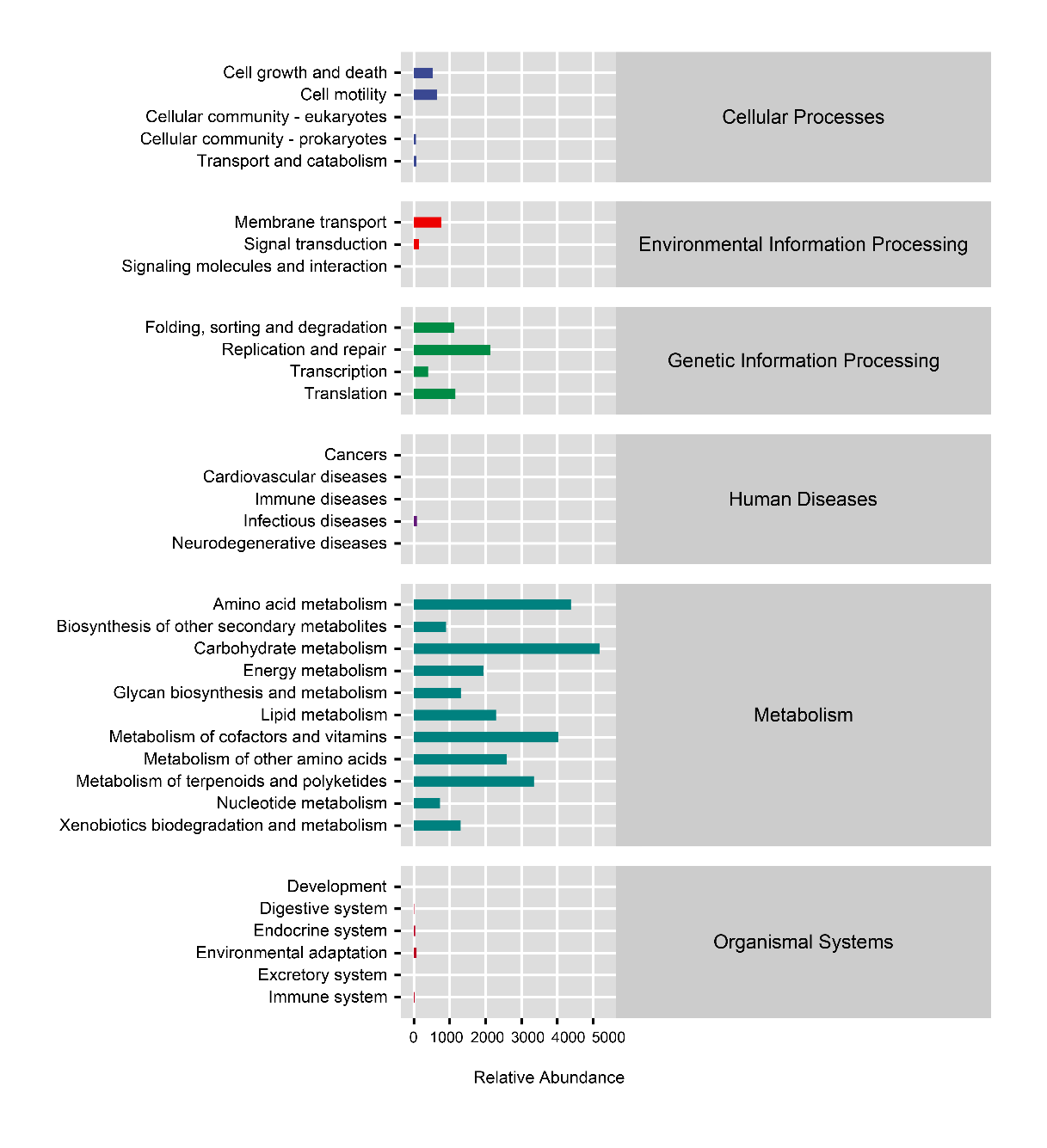


Supplementary Figure 2. Statistical abundance maps of metabolic pathways in the KEGG database of HC and NSCLC groups.


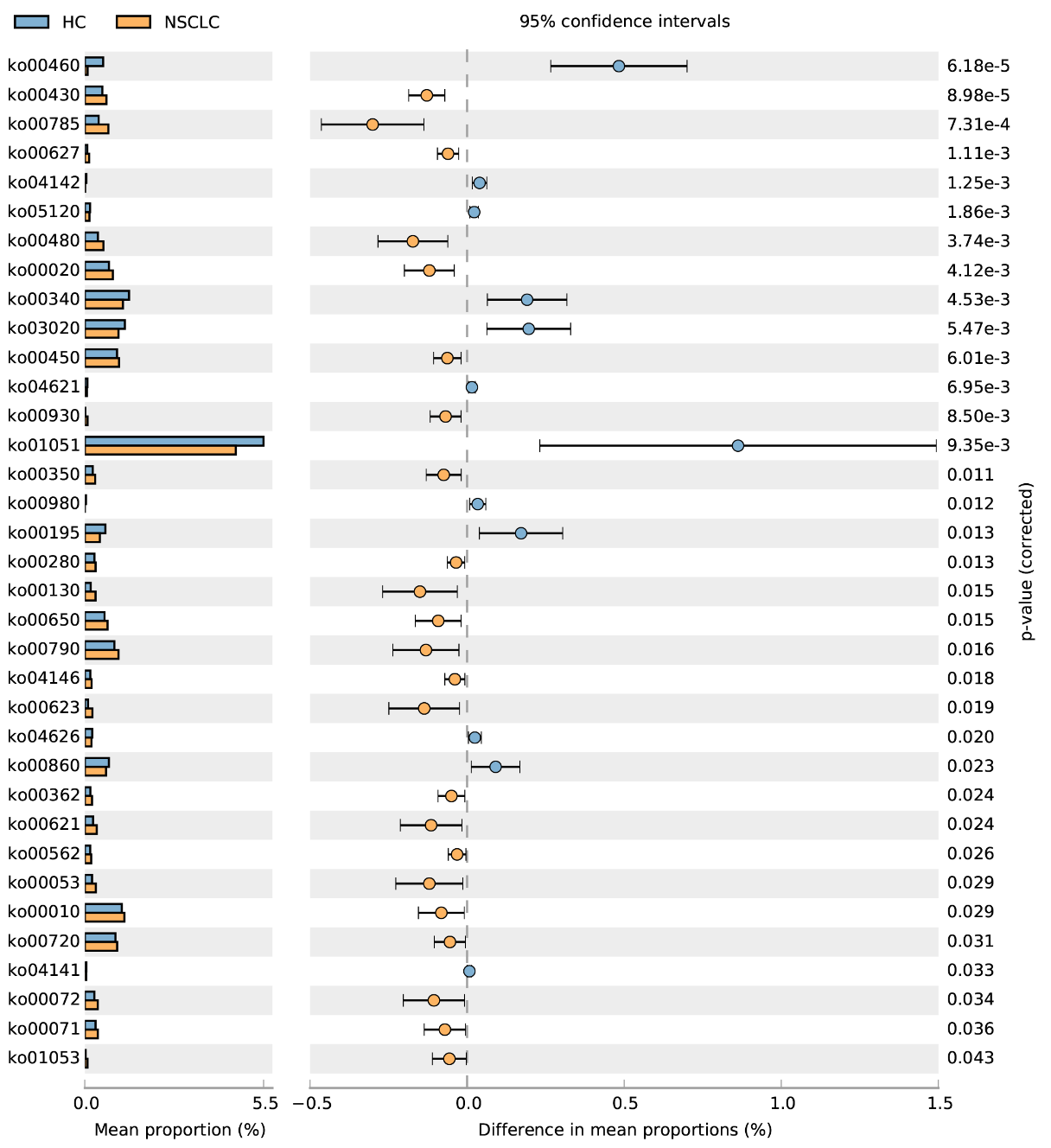


Supplementary Figure 3. Difference analysis of metabolic pathway between NSCLC group and HC group (welch's t-test, all *P* < 0.05).


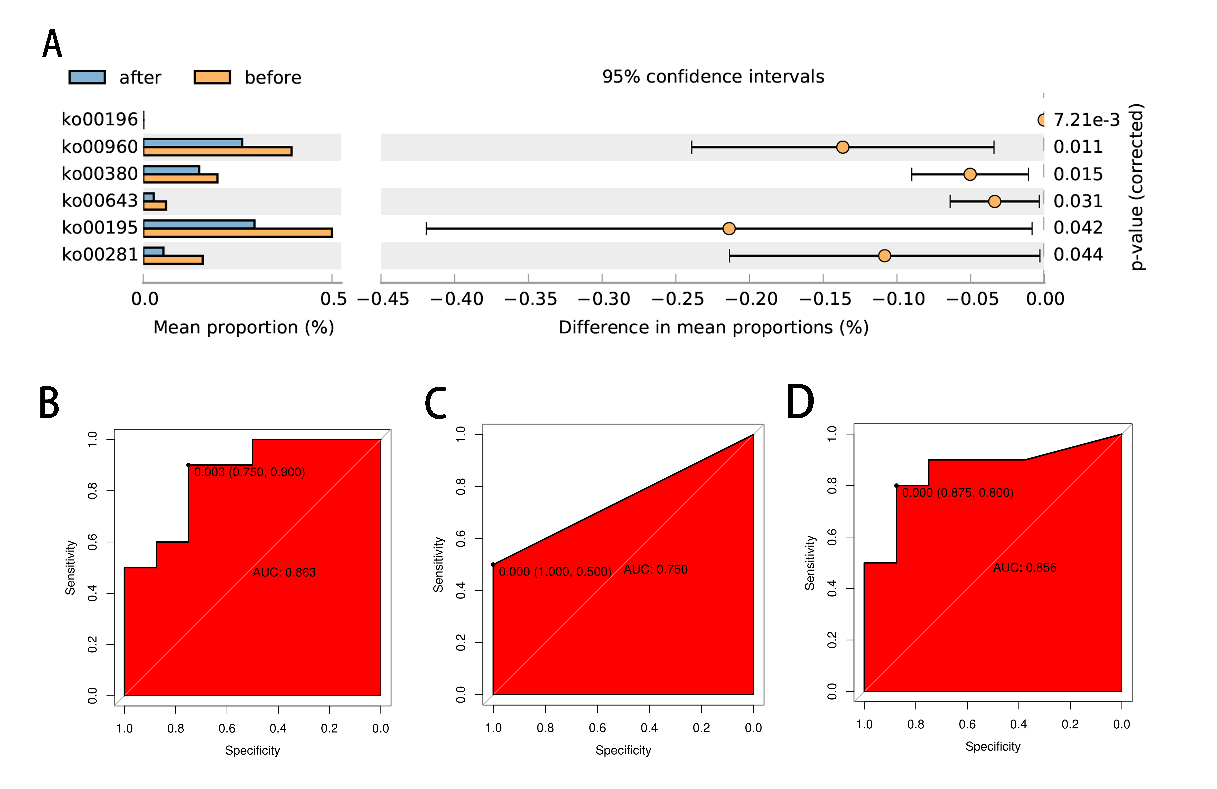


Supplementary Figure 4. Metabolic alterations of gut microbiota in patients undergoing treatment and their prediction of efficacy.

A.Metabolic pathway differences between Before group and After group(welch's t-test, all *P* < 0.05). B.ROC curve of Bifidobacterium predicting the clinical benefit of immunotherapy combined with chemotherapy in NSCLC patients; C. The ROC curve for predicting the clinical benefit of immunotherapy combined with chemotherapy in NSCLC patients; D. Utterella ROC curve for predicting the clinical benefit of immunotherapy combined with chemotherapy in NSCLC patients. Before group: before treatment; After group: after treatment.


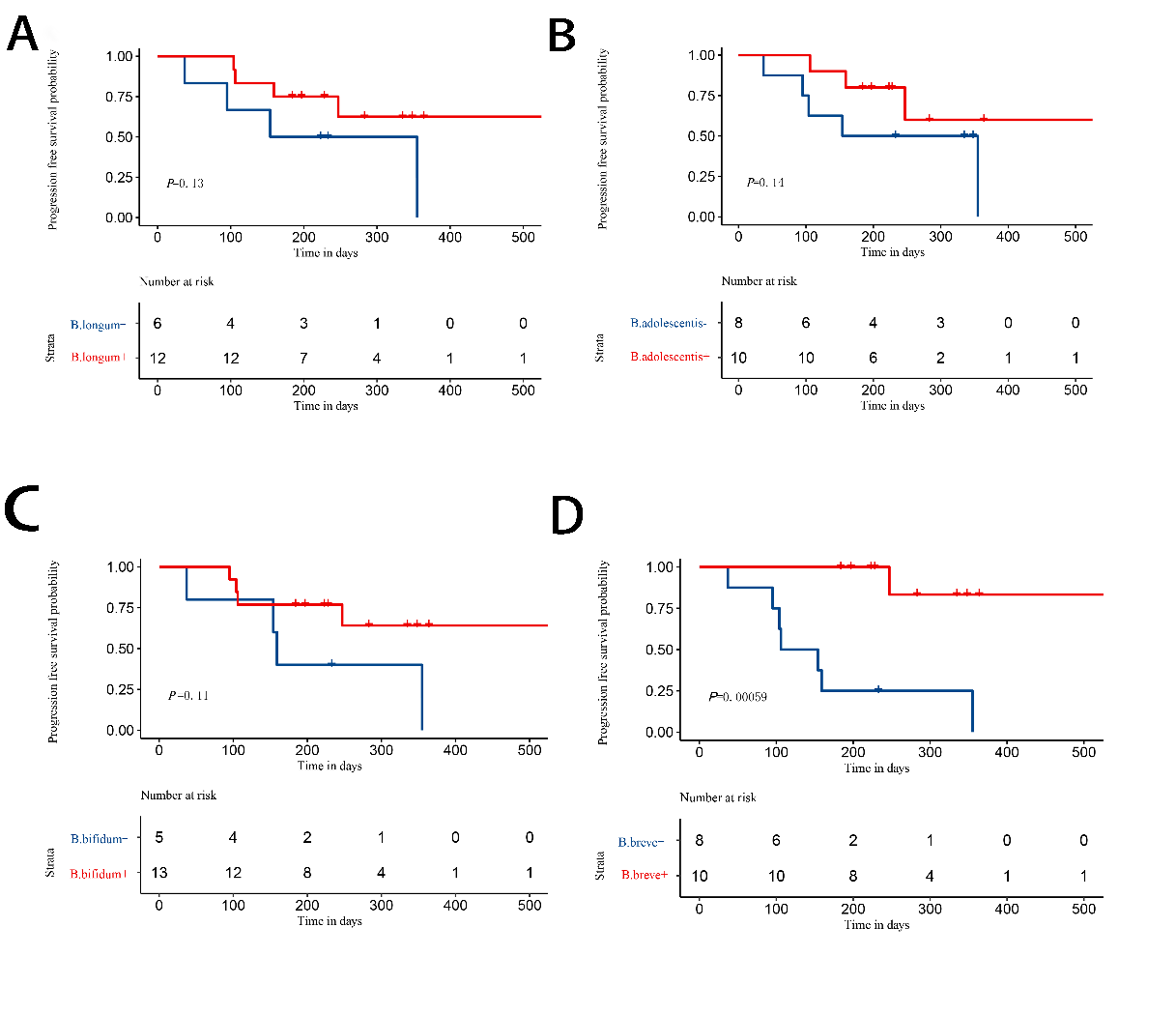


Supplementary Figure 5. Survival analysis of four bacteria.

A. Survival curves and corresponding risk tables of B.longum+ group and B.longum- group(Log Rank test, *P*=0.13). B. Survival curves and corresponding risk tables for B. adolescentis+ group patients and B. adolescentis- group patients(Log Rank test, *P*=0.14) .C. Survival analysis and corresponding risk table of B. bifidum+ group and B. bifidum- group(Log Rank test, *P*=0.011) . D. Survival curves and corresponding risk tables of B. breve+ group and B. breve- group (Log Rank test, *P*=0.000,59).


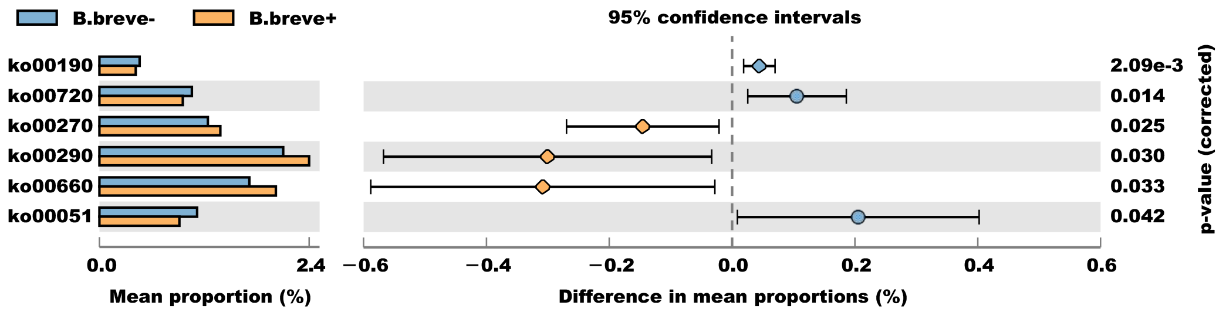


Supplementary Figure 6. Metabolic pathway differences between B. breve+ group and B. breve- group(welch's t-test, all *P* < 0.05).
